# Role of SCO-792, A Novel Enteropeptidase Inhibitor, In the Prevention of Post-Endoscopic Retrograde Cholangiopancreatography Pancreatitis

**DOI:** 10.7759/cureus.13724

**Published:** 2021-03-05

**Authors:** Mohammed Y Rashid, Asfa Noor, Viral Patel, Shereen Henin, Alejandrina Cuello-Ramírez, Anoud S Al kaabi, Anupa Gnawali, Jihan A Mostafa

**Affiliations:** 1 General Surgery, California Institute of Behavioral Neurosciences & Psychology, Fairfield, USA; 2 Research, California Institute of Behavioral Neurosciences & Psychology, Fairfield, USA; 3 Internal Medicine/Pediatrics, California Institute of Behavioral Neurosciences & Psychology, Fairfield, USA; 4 Neonatology, California Institute of Behavioral Neurosciences & Psychology, Fairfield, USA; 5 Family Medicine, California Institute of Behavioral Neurosciences & Psychology, Fairfield, USA; 6 Psychotherapy and Research, California Institute of Behavioral Neurosciences & Psychology, Fairfield, USA

**Keywords:** sco-792, enteropeptidase, enteropeptidase inhibitor, post-endoscopic retrograde cholangiopancreatography pancreatitis, acute pancreatitis

## Abstract

Acute pancreatitis is the most common iatrogenic dilemma of endoscopic retrograde cholangiopancreatography, and it is associated with significant morbidity and mortality. Several factors have been implicated in the pathogenesis of post-endoscopic retrograde cholangiopancreatography pancreatitis, and preventive measures were practiced accordingly. This study aims to refine the potential mechanisms that trigger post-endoscopic retrograde cholangiopancreatography pancreatitis and define the role of enteropeptidase in the pathogenesis of post-endoscopic retrograde cholangiopancreatography pancreatitis. Furthermore, address the role of a new novel medication known as SCO-792, a potent enteropeptidase inhibitor, in the prevention of post-endoscopic retrograde cholangiopancreatography (ERCP) pancreatitis.

Post-endoscopic retrograde cholangiopancreatography pancreatitis is caused by premature activation of the pancreatic enzymes within the pancreatic parenchyma. This activation is either an autoactivation due to direct provocation of intra-acinar enzymes as a result of the procedure or due to activation by enterpeptidase, a rate-limiting enzyme. Endoscopic retrograde cholangiopancreatography interjects duodenal juice that is rich in enterokinase into the pancreatic-biliary tract, which in turn leads to intra-ductal activation of trypsinogen and subsequent enzymes. Given the vital role of enterokinase in initiating the pathogenesis of pancreatitis, enteropeptidase inhibition may prevent and reduce the severity of post-endoscopic retrograde cholangiopancreatography pancreatitis.

SCO-792, a novel enteropeptidase inhibitor, is developed by SCOHIA Pharma, and pre-clinical trials confirmed its efficacy in inhibiting enteropeptidase. Studies are needed to confirm the efficacy of enteropeptidase inhibitors in preventing post-endoscopic retrograde cholangiopancreatography pancreatitis.

## Introduction and background

Post-endoscopic retrograde cholangiopancreatography pancreatitis has been defined as acute pancreatitis occurring 24 h after an endoscopic retrograde cholangiopancreatography procedure, along with the rise of serum amylase level three times the upper limit of normal, necessitating hospitalization [1,2]. Risk factors for post-endoscopic retrograde cholangiopancreatography pancreatitis can be categorized as patient-related factors, procedure-related factors, and operator-related risk factors [3].

Enteropeptidase (enterokinase, EC3.4.21.9) is a transmembrane serine protease at the brush border of the duodenal and jejunal mucosa, and it is responsible for activating pancreatic proteolytic enzymes. Enteropeptidase activates trypsinogen by removing 7-10 amino acids from the N-terminal region known as trypsinogen activation peptide (TAP), and this removal of the TAP induces conformational change resulting in active trypsin, promoting activation of other zymogens and further trypsinogen activation [4-7]. 

Post-endoscopic retrograde cholangiopancreatography pancreatitis is a multifactorial condition, and various methods were implemented to prevent post-ERCP pancreatitis with an acceptable reduction rate. However, none of the interventions could eliminate this complication due to the lack of complete understanding of the disease pathophysiology.

This study aimed to evaluate the role of enteropeptidase in post-endoscopic retrograde cholangiopancreatography (ERCP) pancreatitis. Additionally, appraise the role of a novel medication known as SCO-792 in preventing this complication.

## Review

Annually, around 500,000 endoscopic retrograde cholangiopancreatographies are conducted in the United States. Pancreatitis remains the most common severe complication of endoscopic retrograde cholangiopancreatography, with an incidence rate ranging from 8.8% in average-risk to 14.1% in high-risk patients [8].

Risk factors for post-ERCP pancreatitis

Risk factors that predispose to post-endoscopic retrograde cholangiopancreatography pancreatitis can be classified into three main categories; patient-related factors, procedure-related factors, and operator-related risk factors [8].

Patient-Related Factors

Patient-related factors include the female gender. Although many studies have settled that post-ERCP pancreatitis incidence is higher amongst females, the precise mechanism remains unclear [9,10]. A possible explanation is that sphincter of Oddi dysfunction, which is also an independent risk factor for post-endoscopic retrograde cholangiopancreatography pancreatitis, remains underdiagnosed in females. 

Age (<60 years) is a known risk factor for post-ERCP pancreatitis, and it can be linked to neuro-hormonal reflex. This mechanism is postulated to play a significant role in the pathogenesis of post-ERCP pancreatitis. At the same time, patients older than 60 years are at lower risk for post-ERCP pancreatitis, which can be explained by an age-related neuropathy and atrophy of pancreatic parenchyma [11, 12]. A prior episode of pancreatitis independently raises the risk of post-ERCP pancreatitis [11].

Procedure-Related Risk Factors

Procedure-related risk factors are known as the triggering factor for post-ERCP pancreatitis. It can be explained as any irritative force (trauma, irritation by contrast, and thermal injury) beyond major duodenal papilla, which initiates an inflammatory reaction and subsequently results in pancreatitis by provoking the intra-acinar enzymes. Below are the relevant procedural risk factors.

Cannulation attempts: It has a cumulative effect. A study indicated that the rate of pancreatitis was 3% after easy cannulation (1-5 attempts), 7% after moderately difficult (6-15 attempts), and 13% after difficult cannulation ( >15 attempts) [13]. Performance of biliary sphincterotomy has not been associated with significant added independent risk of pancreatitis in most large-scale prospective multivariate analyses. However, pancreatic sphincterotomy is associated with a variably higher rate of pancreatitis (up to 29%) [13]. 

Contrast injection: It also has a cumulative risk for post-ERCP pancreatitis. A prospective, randomized, controlled trial found the use of the iso-osmolar medium (IOCM) associated with less severe post-ERCP pancreatitis than the usage of high osmolality contrast medium [14]. Thermal injury from electrosurgical devices contributes to the risk of post-ERCP pancreatitis [8,15].

Intubation difficulty and operation time: Both of these act as independent risk factors for post-ERCP pancreatitis. A study indicated that patients with difficult intubation are at higher risk for developing post-ERCP pancreatitis. post-ERCP pancreatitis is also higher when operation time exceeds 60 minutes [16]. 

Operator-Related Risk Factors

Most studies have demonstrated an association between endoscopic retrograde cholangiopancreatography case volume and complication rate. However, these same studies generally have failed to show a significant correlation between endoscopic retrograde cholangiopancreatography case volume and the rate of post-ERCP pancreatitis. Trainee engagement in the procedure is an independent risk factor for post-ERCP pancreatitis [12,17]. 

Mechanism of post-ERCP pancreatitis

**Figure 1:**
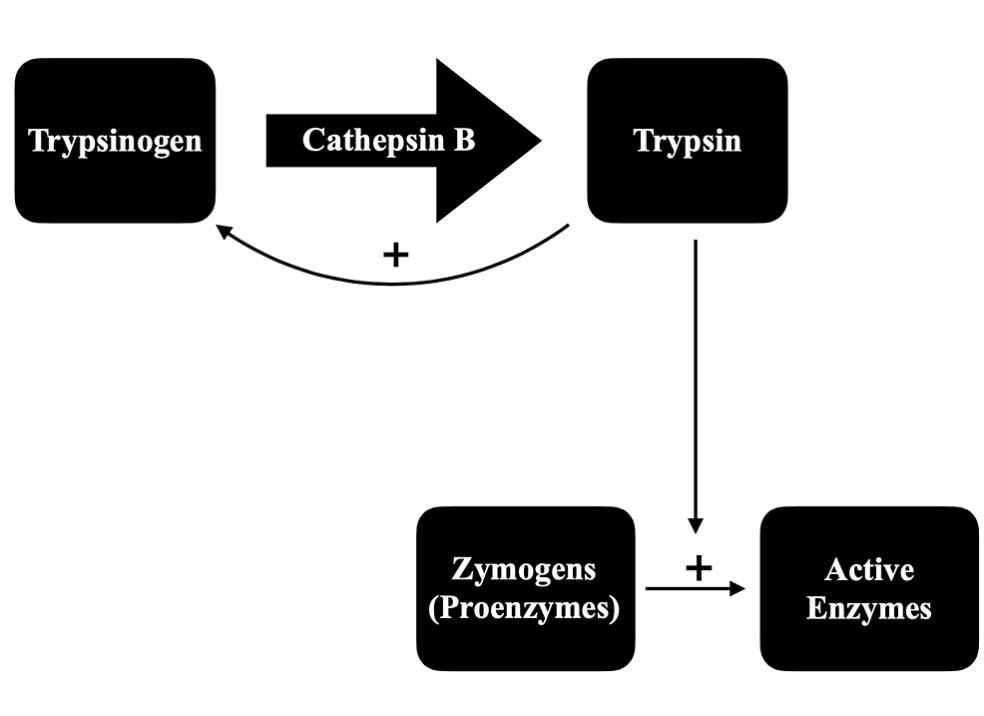
Intra-parenchymal auto-activation of pancreatic enzymes Cathepsin B mediates trypsinogen activation into trypsin. Once trypsin is activated, it will catalyze the activation of other digestive proenzymes as well as trypsinogen itself, thereby autodigestion of the gland [22].

Post-endoscopic retrograde cholangiopancreatography pancreatitis is a multifactorial condition with various postulated mechanisms. Following exposure to the triggering factors, inflammation and injury to the gland occur too rapidly. post-ERCP pancreatitis occurs by one of the following two main mechanisms. The first mechanism is through the theory of intraparenchymal autoactivation of pancreatic enzymes. The irritative force (trauma, irritation by contrast, etc.) results in injury to the pancreatic-biliary tract, which initiates an inflammatory reaction. Thus, it will lead to the accumulation of secretions within the pancreatic duct and the accumulation of zymogens within acinar cells. Subsequently, the lysosome and zymogen will fuse [18-21]. Given this, cathepsin B is a lysosomal enzyme known to activate trypsinogen to trypsin. Once trypsin is activated, it can catalyze the activation of other digestive proenzymes and trypsinogen itself, initiating the gland's autodigestion (Figure 1) [22].

The second mechanism is through the activation of pancreatic enzymes within the pancreatic-biliary tract by enteropeptidase. Normally, pancreatic enzymes are activated by enteropeptidase within the duodenal lumen, not in the pancreatic-biliary tract. However, endoscopic retrograde cholangiopancreatography will interject duodenal juice, which is rich in enterokinase, into the pancreatic-biliary tract. Therefore, this activation will occur within the pancreatic-biliary tract, which causes intrapancreatic activation of trypsinogen, thereby initiating acute pancreatitis. Additionally, intraductal enterokinase markedly increases the pancreatic protein content, probably by increasing the proteolytic enzyme content [23]. As published in multiple studies, slow, low-pressure intraductal injection of enterokinase (EK) in dogs and rats causes hyperamylasemia and acute pancreatitis [24,25]. Given this, enterokinase plays a significant role in initiating the pathogenesis of pancreatitis. 

Furthermore, endoscopic retrograde cholangiopancreatography will also overwhelm the physiologic function of the sphincter of Oddi, and depending on the approach; it may damage the pancreatic duct sphincter and biliary duct sphincter, which increases the risk for future reflux and recurring episodes of pancreatitis.

Preventive methods of post-ERCP pancreatitis

Procedural techniques and pharmacologic interventions have both been studied to reduce the incidence of post-ERCP pancreatitis. The comprehensive review on prevention of post-ERCP pancreatitis published in 2004, a meta-analysis, summarized the various pharmacological and endoscopic interventions that have been implemented in the prevention of post-ERCP pancreatitis. 

The most promising pharmacological agents are Indomethacin, Nitroglycerin, gabexate, and somatostatin. Although gabexate and somatostatin showed an encouraging result, these agents need to be given before the procedure and in a continuous manner for an extended period. Nitroglycerine and Indomethacin are relatively reasonable and straightforward to deliver; however, their efficacy is questionable in patients at high risk for post-ERCP pancreatitis. Additionally, the most promising endoscopic intervention is pancreatic stent placement, especially in high-risk patients. However, it is challenging to define which patient and which procedure guarantees stent insertion [8].

Overall, the prevention of post-ERCP pancreatitis is challenging. Many triggers play a role in a single case of post-ERCP pancreatitis. As a result, each patient needs a thorough preoperative assessment, and a proper preventive approach should be chosen accordingly.

Enteropeptidase 

Enteropeptidase (EC 3.4.21.9) is a critical upstream enzyme in protein digestion. Proenteropeptidase is a precursor for enteropeptidase, which is then activated into a light chain that is a site for catalysis and a heavy chain responsible for anchoring the molecule [26]. Enteropeptidase activates trypsinogen within the duodenal lumen. The activated trypsin will activate the downstream zymogens, which are responsible for the absorption of amino acids and triglycerides [5,27]. Congenital enteropeptidase deficiency in humans is associated with intestinal malabsorption and a lean phenotype, suggesting this enzyme's pivotal role in regulating body homeostasis [28,29]. Studies have proposed that enteropeptidase modulation may be a unique approach for reducing obesity due to the vital role of enteropeptidase in protein and nutrient absorption [30]. Additionally, enteropeptidase plays a critical role in initiating post-ERCP pancreatitis. 

Overview of SCO-792

SCO‐792 (N‐({(3S)‐6‐[(4‐carbamimidamidobenzoyl)oxy]‐2,3‐dihy‐dro‐1‐benzofuran‐3‐yl}acetyl)‐L‐aspartic acid hydrate) is a potent and reversible enteropeptidase inhibitor and exhibits a slow dissociation rate against enteropeptidase. It efficiently inhibits protein digestion, thus, inhibits the elevation of plasma branched-chain amino acid. It also inhibits trypsin activity in vitro [26,31]. Trypsin is a critical downstream molecule of enteropeptidase and thereby it is expected to play a critical role in the management of pancreatitis. 

The enteropeptidase inhibitor SCO-792 efficiently increases insulin sensitivity and glucose control, showing substantial potency in reducing weight. In addition, SCO-792 optimizes plasma and liver lipid profiles [26]. Thus, enteropeptidase inhibition may offer a novel treatment option for obesity, diabetes.

Enteropeptidase inhibitor has therapeutic potential in chronic kidney disease. SCO-792 inhibits glomerular filtration rate decline and suppresses albuminuria. Moreover, SCO-792 improves glomerulosclerosis and suppresses interstitial fibrosis in the kidney [28]. This indicates that SCO-792 treatment is highly effective in improving kidney parameters in patients with chronic kidney disease [31]. 

SCO-792 as a preventive measure for post-ERCP pancreatitis

Enteropeptidase is a specific protease that recognizes and cleaves pancreatic enzymes [32]. As mentioned previously, enteropeptidase plays a vital role in initiating the pathogenesis of pancreatitis, specifically post-ERCP pancreatitis, by introducing duodenal juice that is rich in enterokinase into the pancreatic-biliary tract, in turn, activation of trypsinogen into trypsin within the pancreatic-biliary tract and mediate autodigestion of the gland. SCO-792, a potent enteropeptidase inhibitor, is also a potent protease inhibitor that directly inhibits trypsin [26]. Given this dual mechanism of action, SCO-792 may be a novel and promising prophylaxis and treatment option for pancreatitis.

## Conclusions

Enteropeptidase plays an essential role in the pathogenesis of post-endoscopic retrograde cholangiopancreatography pancreatitis. Thus, enteropeptidase inhibition significantly reduces the rate of post-endoscopic retrograde cholangiopancreatography pancreatitis. SCO-792 is a potent enteropeptidase inhibitor; therefore, it may play a critical role in reducing the incidence of this complication. Additionally, SCO-792 has remarkable therapeutic and preventive advantages in managing obesity, diabetes, kidney and liver function.

This paper adds to the understanding of the underlying mechanism of post-endoscopic retrograde cholangiopancreatography pancreatitis. Future research should focus on clinical trials assessing the efficacy of SCO-792 in preventing post-endoscopic retrograde cholangiopancreatography pancreatitis, as it could be the future management for post-ERCP pancreatitis.
